# Readhesion of Tongue-Tie Following Neonatal Frenotomy: Incidence and Impact of Postoperative Exercises in a Prospective Observational Study

**DOI:** 10.3390/children12080971

**Published:** 2025-07-24

**Authors:** Beatriz Valle-Del Barrio, Silvia Maya-Enero, Jordi Prat-Ortells, María Ángeles López-Vílchez, Júlia Candel-Pau

**Affiliations:** 1 Department of Neonatology, Service of Pediatrics, Hospital del Mar, Parc de Salut Mar, Universitat Pompeu Fabra, Passeig Marítim 25-29, 08003 Barcelona, Spain; bvalle@hmar.cat (B.V.-D.B.); malopez@hmar.cat (M.Á.L.-V.); jcandelpau@hmar.cat (J.C.-P.); 2 Service of Pediatric Surgery, Hospital Sant Joan de Déu, Universitat de Barcelona, Passeig de Sant Joan de Déu, 2, 08950 Esplugues de Llobregat, Spain; joprat@hsjdbcn.org

**Keywords:** ankyloglossia, readhesion, frenotomy

## Abstract

**Background/Objectives:** Frenotomy is the procedure of choice for treating ankyloglossia. The literature reports that readhesion of the frenulum occurs in 2.6–13% of cases. There is no published evidence to support performing tongue exercises to prevent it. We aimed to determine the readhesion rate of ankyloglossia, the benefits of tongue exercises to prevent it, and the characteristics of patients who experienced readhesion. **Methods**: This is a prospective, observational study of neonates who underwent a frenotomy between January and August 2024. Following the frenotomy, we recommended that all parents perform a series of exercises 6–8 times daily over 15 days. Patients were re-evaluated 10–15 days post-procedure for signs of ankyloglossia using the Hazelbaker tool and clinical variables such as nipple pain or cracks. **Results**: We enrolled 212 patients; thirty patients underwent a refrenotomy (14.1%). The raw risk of readhesion in our study was 0.335 (95%CI 0.275–0.401), and for symptomatic readhesion, 0.156 (95%CI 0.113–0.211). Adjusted by sex, the risk of readhesion for female patients was 0.236 (95%CI 0.155–0.344), and for males, 0.390 (95%CI 0.312–0.474). The appearance and function Hazelbaker scores were significantly lower before the frenotomy than post-procedure in all cases. In females, not following the exercise protocol multiplied the risk of readhesion by 1.61 (95%CI 1.03–2.56), whereas in males the risk was multiplied by 1.47 (95%CI 1.03–2.08). Symptomatic readhesion was significantly correlated with age at frenotomy and Hazelbaker score. **Conclusions:** Readhesion of tongue-tie was higher than previously published (33.5%); however, symptomatic readhesion was less frequent (15.6%). Proper adherence to post-frenotomy exercises significantly reduces the risk of readhesion, although it has less impact on symptomatic readhesion.

## 1. Introduction

Ankyloglossia, or tongue-tie (TT), is a condition where a congenital shortened and/or thickened frenulum limits movement of the tongue. It is a common problem in neonates, with an overall estimated incidence ranging between 4 and 16% [1,2,3,4], although some studies have reported a much higher prevalence [5,6]. TT has been recognized as a significant cause of difficulty in establishing breastfeeding and causing distress to both infants and mothers [2,3]. It seems to have a genetic etiology, and, in most cases, symptoms appear in the first three years of life [7].

The standard treatment for ankyloglossia is the frenotomy. Although recent systematic reviews reported no serious complications after frenotomy [8,9,10,11], literature reports that readhesion of the frenulum happens in 2.6–13% of cases [12,13,14,15,16].

There is controversy regarding the need to perform post-frenotomy exercises to decrease the risk of readhesion [4,17,18,19,20,21]. A recent, prospective study found that stretching exercises decreased breastfeeding difficulties subjectively, the development of recurrent frenulum, scarring, and the need for a second frenotomy [22]. Traditionally, we have recommended that all parents perform them post-frenotomy despite the lack of evidence that supports them [4]. Given the lack of universally accepted protocols, exercises are inconsistently described in the literature, with limited high-quality evidence available to select the most suitable care regime [4,23].

The aim of this study was to determine how often ankyloglossia reappears following a frenotomy. As secondary objectives, we analyzed how often parents performed the rehabilitation exercises as recommended and the breastfeeding rates after a frenotomy. Amir et al. found that the Hazelbaker tool has high reliability in assessing infants with tongue-tie and control infants and in recommending a frenotomy [24]. We used the Hazelbaker tool aside from clinical variables such as nipple pain or cracks to measure ankyloglossia.

## 2. Materials and Methods

We conducted a prospective, observational study. Our hospital Ethics Committee (CEIm-PSMAR) approved this study (reference code: 2024/11501) and deemed it unnecessary to obtain written informed consent from the neonates’ parents due to the minimal risk this study involved. However, we verbally informed our patients’ parents that we were conducting this study, what the study entailed, and what we expected of the parents. Patients were only included if their parents gave consent. This study was conducted according to the ethics code of the Barcelona Medical Association and the principles of the Helsinki-Fortaleza Declaration 2013.

This study was conducted at the neonatal unit of a tertiary care hospital in Barcelona (Spain) within an area of influence of approximately 400,000 people, which experiences approximately 1400 births per year. Inclusion criteria were neonates born at Hospital del Mar who had ankyloglossia according to the Hazelbaker tool [25] (Appendix A) or those up to two months of age who were referred to our service for a frenotomy between January and August 2024.

Previous to conducting this study, all attending and nursing staff were trained to evaluate ankyloglossia by using the Hazelbaker tool, with low inter-observer variability. Ankyloglossia is assessed as part of the routine neonatal exam, and breastfeeding support is provided to all mother–neonate dyads. Likewise, we observe the neonate breastfeeding and ask the mother whether she experiences nipple pain and/or if the neonate has difficulty latching on to the breast. We use the LATCH scale to evaluate feeds and the VAS (Visual Analog Scale) scale to quantify maternal nipple pain (0–10). If a lingual frenulum is visible or palpable, a sign of possible TT, either one of the neonatologists or one of the nurses evaluates breastfeeding and uses the Hazelbaker tool to assess its impact on the infant’s tongue movement and on breastfeeding. According to the Hazelbaker tool, ankyloglossia exists if appearance scores 8 points or less and/or function scores 11 points or less. Frenotomy is offered to all tongue-tied patients whose mothers feel pain or who have problems latching on to the breast. However, we do not treat asymptomatic patients. One attending physician trained all staff on how to perform the frenectomy procedure using blunt-tip scissors, and all the frenotomies are performed by one of the three neonatologists or one of the nurses using the technique taught.

During the study, parents were instructed to perform post-frenotomy exercises 6 to 8 times a day, with five repetitions each, over 15 days, and an exercise instruction sheet was provided. The recommended exercises were (1) to stimulate the upper lip with a finger so that the neonate opens the mouth and sticks the tongue out; (2) to touch the lower lip to open the mouth and stick the tongue out; (3) to press both cheeks inwards in order to make the neonate open the mouth and raise the tongue; (4) to move the finger inside the cheek outwards so that he turns the tongue to the side; and (5) to push the tongue towards the palate in order to keep the wound edges separated. This series of exercises has two different goals: exercise #5 aims at opening the wound in case of fibrotic scar tissue formation, whereas the other four exercises intend to stimulate the tongue’s movements to help neuromuscular re-education. All the patients were followed up 10 to 15 days afterward and assessed for breastfeeding ease and ankyloglossia presence using the Hazelbaker tool. Pre- and post-frenotomy scores were compared. Mothers were asked if they experienced nipple pain (but did not quantify it on this occasion) and observed if they had nipple cracks. Symptomatic ankyloglossia was determined if there was still visible/palpable TT, the mother had nipple pain or cracks, and the Hazelbaker tool noted appearance scores of 8 points or less and/or function scores of 11 points or less. Assessment of exercise performance was self-reported by the parents. We evaluated cicatrization 10 to 15 days after the frenotomy because superficial wounds such as that of the frenotomy typically heal within two weeks. We aim not to involve the muscle when performing the frenotomy because such a deep wound is unnecessary, takes longer to heal, and has a higher risk of profuse bleeding due to muscular lesion or accidental section of sublingual veins.

Calculation of sample size: This was intended to be a preliminary study to determine our baseline incidence of readhesion. The estimated incidence of readhesion following a frenotomy, according to the literature, is 2.6 to 13%. We had no previous data on our population; however, we suspected that it was much more common in our population. Since this is purely descriptive and there are no groups to compare, there was no sample size determination. We are currently conducting a clinical trial comparing two groups: an experimental group, where parents perform postoperative exercises, and a control group, where they do not. While we awaited our hospital Ethics Committee’s approval for this clinical trial, we followed all our frenotomized patients to have a basal readhesion incidence that allowed us to determine what sample size would be necessary to draw reliable conclusions in the future clinical trial.

Data collection: We recorded demographic (sex, gestational age, birth weight, and age in hours at the time of frenotomy) and clinical variables (Hazelbaker score pre- and post-frenotomy, whether the participant’s parents had performed the rehabilitation exercises as recommended and how many times per day, whether readhesion existed 10 to 15 days post-frenotomy, need for a second frenotomy, feeding choice, and whether nipple pain existed).

Statistical analysis: Quantitative variables (gestational age, birth weight, and age at frenotomy) are described using the mean, standard deviation, and range, and compared using a Student’s *t*-test. Sex is described with percentages and compared with a Fisher’s exact test. Hazelbaker scores pre- and post-frenotomy are described using the median and interquartile range (IQR) and compared with a Wilcoxon sum-rank test. Readhesion of the tongue-tie, presence of symptoms post-frenotomy, type of feeding, and need for a second frenotomy are presented in percentages and compared using a Fisher’s exact test. We established a logistic regression model using potential readhesion predictors such as gestational age, sex, birth weight, age at frenotomy, and exercise adherence. Statistical significance was set at *p* < 0.05. To perform the statistical analyses, we used STATA version 16.1 (StataCorp., College Station, TX, USA).

## 3. Results

**Figure 1:**
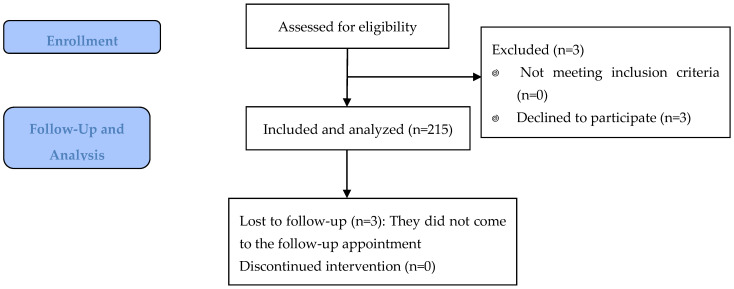
CONSORT 2010 Flow Diagram.

We enrolled 215 patients from a total of 218 potential candidates between 16 January and 5 August 2024. Three parents refused to participate and, of the 215, three patients were lost to follow-up. Thus, 212 patients were included in the analysis (see Figure 1).

### 3.1. Description of Our Population

We included 138 male (64.2%) and 77 female (35.8%) newborns. Mean (SD) gestational age was 39^2/7^ (1^6/7^) weeks (range: 30^0/7^–42^2/7^ weeks) and mean (SD) birth weight was 3231.4 (535.4) g (range: 1284–4650 g). The median (IQR) age at the time of the procedure was 59 (229) hours (range: 5–1440 h). Table 1 shows the demographic characteristics of the readhesion and non-readhesion groups.

### 3.2. Feeding

Most neonates were exclusively breastfed (142/212, 67.0%), whereas 61/212 were partially breastfed (28.8%). Nine received formula only (4.2%). Table 2 shows the type of feeding depending on the presence of readhesion or not, and in the case of readhesion, depending on symptomatic vs. asymptomatic readhesion.

### 3.3. Hazelbaker Scores

**Figure 2:**
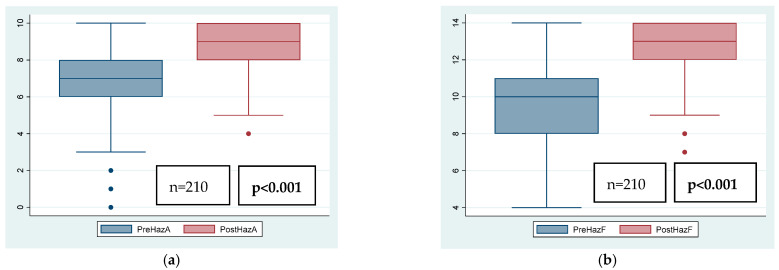
Global Hazelbaker score for appearance (A) and function (F) pre- and post-frenotomy. (**a**) PreHazA: Hazelbaker score for appearance before frenotomy; PostHazA: Hazelbaker score for appearance after frenotomy; (**b**) PreHazF: Hazelbaker score for function before frenotomy; PostHazF: Hazelbaker score for function after frenotomy.

Median (IQR) Hazelbaker scores pre-procedure were 6.4 (1.8) for appearance and 9.3 (1.7) for function, and post-procedure 8.9 (1.2) for appearance and 12.5 (1.6) for function (*p* < 0.001). As expected, mean Hazelbaker scores both for appearance and for function were lower before the frenotomy than after, regardless of the presence of readhesion or symptoms post-frenotomy. Median Hazelbaker score increase following frenotomy was two points (95% confidence interval (CI): 2–3) for appearance and three points (95%CI: 3–3.7) for function (*p* < 0.001). Table 3 shows the outcomes after frenotomy depending on the presence or absence of readhesion. Figure 2 shows global Hazelbaker scores for appearance and function pre- and post-frenotomy. The Hazelbaker scores for appearance and function and their increase post-frenotomy were significantly lower when symptoms were still present after the frenotomy.

### 3.4. Readhesion

The raw risk of readhesion in our study was 0.335 (95%CI 0.275–0.401%) (see Table 4), and for symptomatic readhesion, 0.156 (95%CI 0.113–0.211). However, major differences appeared by sex. The same pattern is observed in symptomatic readhesion. Adjusted by sex, the risk of readhesion is as follows:

- Female patients: global readhesion risk = 0.236 (95%CI 0.155–0.344); symptomatic readhesion risk = 0.105 (95%CI 0.543–0.194)
- Male patients: global readhesion risk = 0.390 (95%CI 0.312–0.474); symptomatic readhesion risk = 0.183 (95%CI 0.128–0.257)

It is interesting to observe that the relative risk (RR) of readhesion for male patients compared to females is 1.65 (95%CI 1.04–2.59), *p* = 0.0237. Regarding symptomatic readhesion, this RR is 1.75 (95%CI 0.83–3.68), which is not statistically significant (*p* = 0.1303), possibly due to the low incidence of symptomatic readhesion.

### 3.5. Patients at Risk for Readhesion

**Figure 3:**
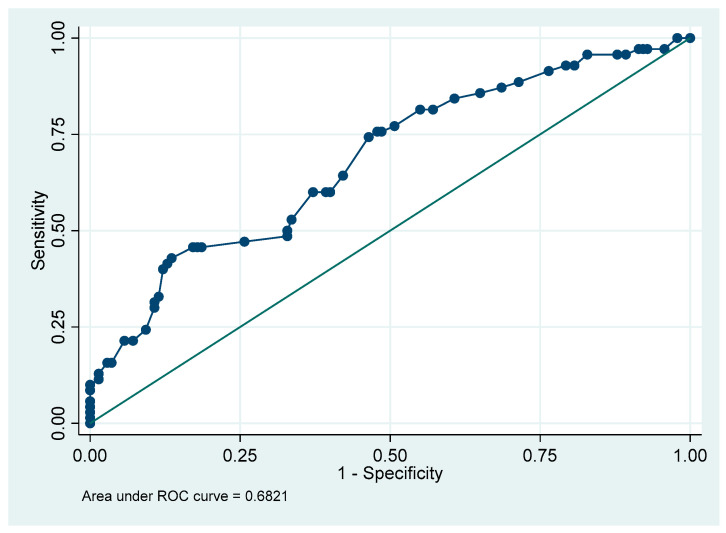
Model ROC curve.

We recommended that all parents do post-frenotomy exercises. Eleven families did not do them at all (5.2%), 76 did them but less frequently than recommended (35.8%), and 125 performed them as recommended (6 to 8 times per day over 15 days) (59.0%). We modeled a logistic regression to estimate the risk of readhesion based on adherence to the protocol. To create the model, we performed a backward stepwise logistic regression based on the degree of significance. In addition, we computerized all possible models (63 models) including all predictor variables and their interactions, selecting the best model according to its AIC, AUC, and simplicity. Finally, both methods provided a prediction model that considered the variables “sex”, “exercises”, and “total Hazelbaker score”. The model has an area under the curve of AUC = 0.68 (95% CI: 0.61 to 0.74, calculated according to Wilson’s exact method). Figure 3 shows the model ROC curve. There is no collinearity in the model (mean VIF = 1).

**Figure 4:**
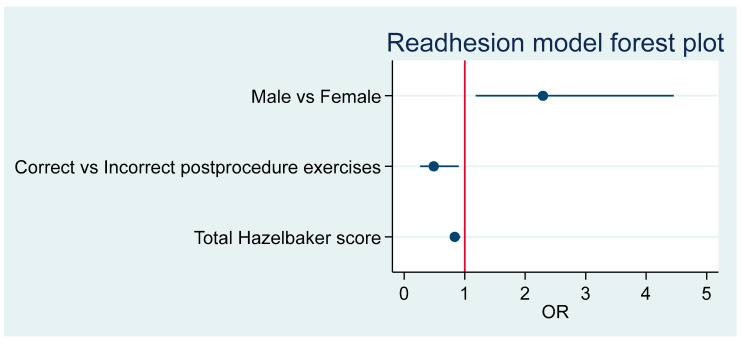
Summary of predictor effects.

The male odds ratio (OR) for readhesion compared to females is 2.29 (95%CI 1.18–4.48), *p* = 0.014. Post-frenotomy adherence to protocol is associated with readhesion, OR = 0.49 (95%CI 0.26–0.90), *p* = 0.022. An increase of one unit in total Hazelbaker score is associated with readhesion at an OR = 0.84 (95%CI 0.75–0.93), *p* = 0.001. Figure 4 summarizes these results.

According to this model, the adjusted RR of readhesion for those who follow the protocol exercises and have the mean average Hazelbaker score of 16 is for males RR = 0.64 (95%CI 0.44–0.94, *p* = 0.022), whereas for females RR = 0.57 (95%CI 0.35–0.93, *p* = 0.023). In other words, among females with Hazelbaker score of 16, not following the exercise protocol multiplies the risk of readhesion by 1.75 (95%CI 1.08–2.85); however, in male neonates with Hazelbaker score of 16, not following the exercise protocol multiplies the risk of readhesion by 1.55 (95%CI 1.07–2.26).

### 3.6. Symptomatic Readhesion

There were 33 patients who had a symptomatic readhesion, of which thirty underwent a second frenotomy. We analyzed the group of patients with symptomatic readhesion, to whom we always offered a refrenotomy (that was performed provided the parents consented to it).

There were only two variables that differed between those who experienced symptomatic readhesion and those who did not: age at frenotomy (measured in hours) and the sum of Hazelbaker scores (appearance and function). The average age for the non-readhesion group was 173 h vs. 333 h for the adhesion group (*p* = 0.0351).

We created a logistic regression model to identify the main predictors of readhesion in our population. The model was selected according to the criteria of minimum AIC and maximum AUC, analyzing all 63 possible models, including interactions between variables and following a hierarchical principle. The best model for symptomatic readhesion is the one that includes the predictors sex, age at frenotomy (hours), total Hazelbaker score, and exercise performance. There are no collinearity issues in this model (mean VIF = 1.02), despite the fact that the variables age at frenotomy and total Hazelbaker score show a low, albeit significant, linear correlation (r = 0.137, 95%CI of r: 0.003 to 0.267).

**Figure 5:**
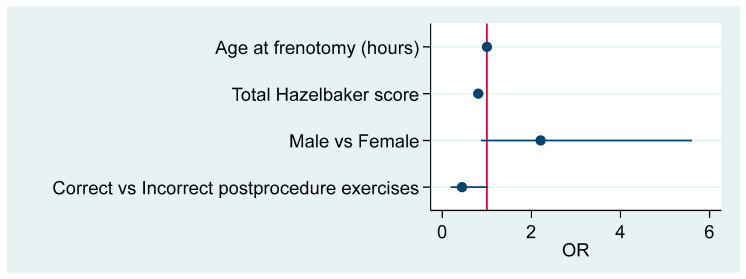
Summary of the model’s effects.

Table 5 shows the OR associated with the model, which is significant (*p* = 0.0001) and has a pseudo R^2^ = 0.1339. Figure 5 offers the forest plot associated with this model.

According to this model, higher risk for symptomatic readhesion and need for a second frenotomy corresponded to male patients who underwent a late frenotomy, had a lower (more pathological) Hazelbaker score, and did not follow the postoperative exercise regime.

The predictive capacity of this model has an area under the ROC curve of 0.77 (95%CI 0.71–0.83). The optimal cutoff point is that at which the model offers a *p* = 0.341. This cutoff has a low sensitivity of 29.0% (90%CI 16.1–46.6, calculated according to Wilson’s exact method) but a high specificity of 96.1% (95%CI 92.1–98.1%, Wilson), which provides a positive predicted value of 56.2% and a negative predictive value of 88.6%.

## 4. Discussion

Several authors have reported the benefits of performing a frenotomy to treat TT [1,3,23,26,27] but few reported the incidence of readhesion after this procedure [12,13,14,15,16,22]. Most of these studies only reported the overall low incidence of readhesion and did not mention which criteria they used to describe readhesion. Furthermore, none of the authors mentioned the incidence of refrenotomy due to readhesion or the presence of asymptomatic readhesion (no nipple pain or cracks and correct weight gain). Recently, Miller et al. conducted a prospective cohort study where some patients were assigned to a stretching group (and had to perform post-operative exercises) and the others to a non-stretching group. They evaluated patients a month later and observed that breastfeeding difficulties, the development of recurrent frenulum, scarring, and the need for a second frenotomy improved in the stretching group. In Miller’s study, only 57.1% of the families performed the exercises as instructed (by placing two fingers underneath the patient’s tongue and stretching the tip of the tongue to the hard palate five times daily, for either two or six weeks). Recurrent ankyloglossia overall affected 19.3% of their patients: 5.6% in the stretching adherent group, 40.7% in the stretching non-adherent group, and 16% in the non-stretching group. A revision procedure was necessary in 22.7% of their patients (5.6, 37.0, and 32%, respectively). However, their population was not neonatal but 50 days old on average [22].

We had the purpose of performing a randomized clinical trial to determine the effect of postoperative exercises on readhesion. However, we did not know the real readhesion rate of our population; therefore, this study had the main purpose of estimating our readhesion rate, defining it as the presence of a visible and/or a palpable tongue-tie as well as a pathological Hazelbaker score. We considered readhesions symptomatic when, in addition to a visible and/or palpable tongue-tie and a pathological Hazelbaker score, there were accompanying symptoms such as pain while breastfeeding, problems latching on to the breast, and poor weight gain. By contrast, we considered readhesions asymptomatic when the visible and/or palpable tongue-tie with a pathological score was not accompanied by symptoms. This study confirmed that the overall incidence of readhesion post-frenotomy in our population is much higher than previously reported, 33.5% globally. We did not perform a refrenotomy in case of asymptomatic readhesions but recommended follow-up as well as physiotherapy/osteopathy sessions. The incidence of symptomatic readhesion requiring a refrenotomy was lower at 15.6%, similar to that reported in the literature.

Several authors have reported that undergoing a frenotomy in combination with different types of pre- and postoperative care was associated with low ankyloglossia recurrence rates and only minor postoperative complications [7,11,28]. Some authors suggested that higher rates of readhesion could be linked to the presence of posterior tongue-ties [13] and the lack of exercises. According to Ferrés-Amat et al. [7], it is important to perform orofacial myofunctional rehabilitation following surgical treatment for ankyloglossia. In Frezza et al.’s review of the literature, 96% of the patients who underwent a frenotomy and myofunctional rehabilitation to improve lingual mobility, both prior to surgical therapy and in the postoperative period, improved [29]. Furthermore, contrary to what other authors found, we observed a lower incidence of readhesion when postoperative exercises were correctly performed (28%). It is important to note that the target populations of the previously presented studies were not exclusively neonatal, as in our case. We believe that active wound management (that is, performing some kind of massage directly on the surgical wound to prevent readhesion), in the form of stretching and strengthening exercises, may contribute to effective healing and reduce reattachment, leading to improved function and tongue mobility.

The goals of performing a frenotomy are to achieve no pain or minimal pain for the mother, adequate weight gain for the neonate/infant with exclusive breastfeeding, and efficient feeding. Our study showed a significant decrease in painful breastfeeding after frenotomy. We observed that none of the neonates had symptoms post-frenotomy unless readhesion existed, whereas 46.5% of the neonates who suffered readhesion continued to have symptoms after frenotomy. Only 18.4% of the mothers reported nipple pain at the follow-up appointment post-frenotomy, and 95.3% of the neonates were breastfed: 66.7% were exclusively breastfed, with an additional 28.6% who were partially breastfed. Thus, in the vast majority of cases, performing a frenotomy improved breastfeeding, which is the main goal of performing this procedure.

This study has many strengths. First, it is a prospective, observational study with a large sample size. Second, it examines readhesion according to a validated, objective tool, the Hazelbaker tool, which is used worldwide. However, the originality of our study lies in the application and interpretation of its results, not in the tool itself. Finally, to the best of our knowledge, this is the first prospective study to introduce the concepts of symptomatic and asymptomatic readhesion. We believe that future research should focus on monitoring these patients to evaluate the potential clinical implications of such findings. According to our results, performing postoperative exercises appropriately is associated with lower readhesion rates.

We acknowledge that this study also has some limitations. First, it is single-center, which may limit its external validity. In order to maximize external validity and adaptation to real-life situations, an intention-to-treat analysis is the standard. However, as this was an observational study and all families were instructed to follow the same postoperative exercise regime, only per-protocol analysis is possible. Second, we did not quantify whether the parents understood the performance of all the post-procedure exercises, even though we demonstrated them and had the parents demonstrate them back prior to discharge. Third, we relied on our patients’ parents’ honesty when determining how often the exercises were performed at home. Fourth, we assessed the performance of all the post-surgery exercises assuming that all of them were equally important. Compliance with the exercises was low: only 59.4% of the families performed them as recommended. The exercise frequency may be too high, leading to less compliance. Consistently with this, we observed that 35.8% of the families performed the exercises with a lower frequency than recommended. We also acknowledge that there could be confounding factors that we could not control, such as the parents’ interest in performing the postoperative exercises and previous experiences, and others that could have affected our findings. In the future, we could analyze if recommending less frequent performance of exercises leads to a higher compliance without a higher readhesion rate, as some authors suggest [4,7,20,28,30,31,32,33,34]. In order to externally validate our findings and improve the management of ankyloglossia, further multicenter studies are required. Future research should focus on the impact of asymptomatic readhesion and post-surgery exercises and consider additional factors, such as determining if any of the exercises are more important than others.

Currently, published evidence on the incidence of post-frenotomy readhesion is scarce, and there are no available guidelines regarding the follow-up management of pathological TT. Many studies agree on the usefulness of incorporating post-surgical rehabilitation exercises to decrease the incidence of readhesion. Nonetheless, it is desirable to conduct further in-depth studies on this topic, especially those focused on the neonatal population. As a future direction, we believe that providing our patients’ parents with video guides, AI tools, or apps to help them perform the postoperative exercises could enhance compliance with their correct performance.

Logistic regression models are mathematical models that try to explain results according to some predictors. To simplify the model, we treated the Hazelbaker score as a continuous variable, although in a strict sense it is not. However, we believe that we can make this assumption, as the exact mathematical results are not as important as the basic ideas behind the model.

In our series, sex was a key predictor in readhesion. This idea has not been published before. Being female acted as a protective factor against readhesion. We cannot hypothesize a reason for this finding. Even though sex was not significant in the symptomatic readhesion model, it was important in the general model. Thus, we consider it worth mentioning.

Age at frenotomy and Hazelbaker score were significant predictors of symptomatic readhesion. Post-frenotomy exercises help to reduce the readhesion rate. However, its importance is lower for symptomatic readhesion, although they are also beneficial. Some parents complain that exercises are painful. Further studies are needed to target these exercises exclusively to the patients/neonates that may benefit the most from performing them.

## 5. Conclusions

The incidence of readhesion post-frenotomy in our population was 33.5% overall. Our data suggest that diligent performance of postoperative tongue exercises reduced the incidence of tongue-tie readhesion and may have also reduced symptoms, thus improving neonatal breastfeeding outcomes. Sex was also linked with readhesion; males’ OR of readhesion was double that of females. These findings underscore the need for structured postoperative protocols. As expected, the Hazelbaker scores were lower pre-frenotomy than post-frenotomy, and they continued to be so regardless of the performance of such exercises.

## Figures and Tables

**Table 1:** Demographic characteristics of the readhesion and non-readhesion groups.

	Readhesion n = 71 (33.5%)	No Readhesion n = 141 (66.5%)	*p*-Value
Male newborn (n, %)	53 (39.0)	83 (61.0)	0.033 ^a^
Birth weight (grams), mean (SD) (range)	3252.6 (482.2) (1284–4175)	3221.1 (560.8) (1640–4650)	0.70 ^b^
Gestational age (weeks), mean (SD) (range)	39^1/7^ (1^6/7^) 30^0/7^–42^0/7^	39^2/7^ (1^6/7^) 30^5/7^–42^2/7^	0.92 ^b^
Age at frenotomy (hours) median (IQR) (range)	50 (308) (5–1440)	61 (176) (15–1152)	0.40 ^b^

^a^ Fisher’s exact test, ^b^ Student’s *t*-test.

**Table 2:** Breastfeeding after frenotomy depending on the presence or absence of readhesion.

	Exclusive Breastfeeding	Partial Breastfeeding	*p*-Value ^a^
No readhesion (n, %)	92 (64.79)	43 (30.28)	0.07
Asymptomatic readhesion (n, %)	31 (83.78)	4 (10.81)	
Symptomatic readhesion (n, %)	19 (55.88)	14 (41.18)	
Total	142 (66.67)	61 (28.64)	

^a^ Fisher’s exact test.

**Table 3:** Outcomes after frenotomy depending on the presence or absence of readhesion.

	Readhesion n = 71 (33.5%)	No Readhesion n = 141 (66.5%)	*p*-Value
Symptoms			
Yes (n, %)	33 (46.5)	0 (0)	-
No (n, %)	38 (53.5)	0 (0)	
Hazelbaker score for appearance pre-frenotomy			
median (IQR)	6 (3)	7 (2)	<0.001 ^a^
(range)	(1–10)	(0–10)	
Hazelbaker score for appearance post-frenotomy			
median (IQR)	8 (2)	10 (1)	<0.001 ^a^
(range)	(4–10)	(8–10)	
Increase in Hazelbaker score for appearance pre- vs. post-frenotomy, mean (SD)	2.3 (2.0)	2.7 (1.6)	0.106 ^b^
Hazelbaker score for function pre-frenotomy			
median (IQR)	9 (28)	10 (3)	0.005 ^a^
(range)	(4–12)	(5–14)	
Hazelbaker score for function post-frenotomy			
median (IQR)	11 (3)	13 (2)	<0.001 ^a^
(range)	(7–14)	(10–14)	
Increase in Hazelbaker score for function pre- vs. post-frenotomy, mean (SD)	2.3 (2.4)	3.7 (1.9)	<0.001 ^b^

^a^ Bonett–Price median comparison test, ^b^ Student’s *t*-test.

**Table 4:** Presence of readhesion following a frenotomy depending on the performance of lingual exercises.

	Readhesion n = 71 (33.5%)	No Readhesion n = 141 (66.5%)	*p*-Value ^a^
No exercises (n, %)	8 (72.7)	3 (27.3)	0.017
Exercises incorrectly done (n, %)	28 (36.8)	48 (63.2)	
Exercises correctly done (n, %)	35 (28.0)	90 (72.0)	

^a^ Chi square test.

**Table 5:** Predictor risk factors for symptomatic readhesion.

	OR	95%CI	*p*-Value
Sex ^a^	2.207	0.869–5.606	0.096
Age at frenotomy (hours)	1.002	1.001–1.004	0.000
Total Hazelbaker score	0.807	0.706–0.923	0.002
Exercices ^b^	0.443	0.190–1.030	0.059

^a^ Female sex is the category of reference; ^b^ Non-adherence to protocol is the category of reference.

## Data Availability

The data presented in this study are available on request from the corresponding author due to our study data not being in a public repository.

## Abbreviations

The following abbreviations are used in this manuscript:

AIC	Akaike information criterion
AUC	Area under the curve
CI	Confidence interval
IQR	Interquartile range
OR	Odds ratio
ROC	Receiver Operating Characteristic
RR	Relative risk
SD	Standard deviation
TT	Tongue-tie

## Appendix A

**Table A1:** Hazelbaker Assessment Tool for Lingual Frenulum Function (HATLLF).

Hazelbaker Assessment Tool for Lingual Frenulum Function (HATLLF)			
Appearance items	Score	Function items	Score
Appearance of tongue when lifted		Lateralization	
Round or square	2	Complete	2
Slight cleft in tip apparent	1	Body of tongue but not tongue tip	1
Heart-shaped	0	None	0
Elasticity of frenulum		Lift of tongue	
Very elastic (excellent)	2	Tip to mid-mouth	2
Moderately elastic	1	Only edges to mid-mouth	1
Little or no elasticity	0	Tip stays at alveolar ridge or rises to mid-mouth only with jaw closure	0
Length of lingual frenulum when tongue lifted		Extension of tongue	
More than 1 cm or embedded in tongue	2	Tip over lower lip	2
1 cm	1	Tip over lower gum only	1
Less than 1 cm	0	Neither of above, or anterior or midtongue humps	0
Attachment of lingual frenulum to tongue		Spread of anterior tongue	
Posterior to tip	2	Complete	2
At tip	1	Moderate or partial	1
Notched tip	0	Little or none	0
Attachment of lingual frenulum to inferior alveolar ridge		Cupping	
Attached to floor of mouth or well below ridge	2	Entire edge, firm cup	2
Attached just below ridge	1	Side edges only, moderate cup	1
Attached at ridge	0	Poor or no cup	0
Total appearance score		Peristalsis	
Function items score 14: perfect score (regardless of Appearance item score) 11: acceptable, if Appearance item score is 10 <11: function impaired Frenotomy should be considered if management fails. Frenotomy necessary if Appearance item score is <8.		Complete, anterior to posterior (originates at the tip)	2
		Partial: originating posterior to tip	1
		None or reverse	0
		Snapback	
		None	2
		Periodic	1
		Frequent or with each suck	0
		Total function score	
